# Increased differentiation associates with decreased polyfunctionality for HIV but not CMV-specific CD8+ T cell responses

**DOI:** 10.1186/1742-4690-9-S2-P250

**Published:** 2012-09-13

**Authors:** C Riou, M Abrahams, K Mlisana, R Koup, M Roederer, S Abdool Karim, G de Bruyn, C Williamson, CM Gray, WA Burgers

**Affiliations:** 1 University of Cape Town, Cape Town, South Africa; 2 Division of Virology, University of Cape Town, Cape Town, South Africa; 3 CAPRISA and Medical Microbiology Dept, University of KwaZulu Natal, Durban, South Africa; 4 Vaccine Research Center, NIH, Bethesda, MD, USA; 5 CAPRISA, University of KwaZulu Natal, Durban, South Africa; 6 Perinatal HIV Research Unit, Chris Hani Baragwanath Hospital, Johannesburg, South Africa

## Background

The generation of polyfunctional CD8+ T cells, in response to vaccination or natural infection, has been associated with improved protective immunity. However, it remains unclear whether the maintenance of polyfunctionality is linked to particular phenotypic characteristics of the cell, such as the differentiation stage of memory T cells. The goal of this study was to investigate the relationship between the memory maturation stage and polyfunctional profiles of antigen-specific CD8+ T cells.

## Methods

We analyzed the polyfunctionality of HIV-specific CD8+ T cells within different memory subpopulations in 20 ART-naïve HIV-1 infected individuals at approximately 34 weeks post-infection and compared them to CMV-specific CD8+ T cell responses. Memory subsets were distinguished based on CD45RO and CD27 expression levels, and four functions were assessed (CD107a, MIP-1β, TNFα and IFNγ).

## Results

Our results show that polyfunctional abilities of HIV-specific CD8+ T cells differ according to their memory phenotype, where terminally-differentiated HIV-specific CD8+ T cells (CD45RO-CD27-) were mostly mono-functional (median 69% [IQR: 57-83]), producing predominantly CD107a or MIP-1β. Moreover, the proportion of HIV-specific mono-functional CD8+ T cells associated positively with the proportion of terminally-differentiated HIV-specific CD8+ T cells (p=0.019, r=0.54). On the other hand, HIV-specific early-differentiated cells of a central memory-like phenotype (CD45RO+CD27+) exhibited a higher proportion of cells positive for three or four functions (p<0.001) and a lower proportion of mono-functional cells (median 27% [IQR: 16-38], p<0.001) compared to terminally-differentiated cells. In contrast, CMV-specific CD8+ T cell polyfunctional capacities were similar across all memory subpopulations, with terminally and early-differentiated cells endowed with comparable polyfunctionality.

## Conclusion

Overall, these data show that for HIV-specific responses, the memory differentiation stage can influence the polyfunctional properties of CD8+ T cells, suggesting that terminal differentiation of HIV-specific CD8+ T cells might be detrimental for viral control. These results may help in understanding phenotypic attributes related to effective T cell responses against HIV-1.

